# Case report: Catecholamine cardiomyopathy in children with neuroblastoma

**DOI:** 10.3389/fped.2023.1063795

**Published:** 2023-02-09

**Authors:** Xiaoyan Xu, Meiqi Liu, Yanmin Zhang, Jie Wang, Xi Lei, Juanli Wang, Yafei Zhou, Tao Wang

**Affiliations:** ^1^ Department of Cardiology, Xi’an Children’s Hospital, Affiliated Children’s Hospital of Xi’an Jiaotong University, Xi’an, China; ^2^ National Regional Children’s Medical Center (Northwest), Key Laboratory of Precision Medicine to Pediatric Diseases of Shaanxi Province, Xi’an Key Laboratory of Children’s Health and Diseases, Shaanxi Institute for Pediatric Diseases, Xi’an Children’s Hospital, Affiliated Children’s Hospital of Xi’an Jiaotong University, Xi’an, China; ^3^ Institute of Children’s Diseases, Xi’an Children’s Hospital, Affiliated Children’s Hospital of Xi’an Jiaotong University, Xi’an, China

**Keywords:** catecholamine cardiomyopathy, neuroblastoma, children, hypertrophic cardiomyopathy, hypertension

## Abstract

**Introduction:**

Many endocrine diseases, such as neuroblastoma (NB), can be linked with acquired cardiomyopathy and heart failure. Neuroblastoma’s cardiovascular manifestations are typically hypertension, electrocardiogram (ECG) changes, and conduction disturbances.

**Case Presentation:**

A 5-year-old 8-month-old girl was admitted to the hospital with ventricular hypertrophy and hypertension (HT) and heart failure. She had no previous history of HT. On color doppler echocardiography, the left atrium and left ventricle were enlarged. The left ventricular ejection fraction (EF) was as low as 40%, and the ventricular septum and left ventricular free wall were thickened. The internal diameters of both coronary arteries were widened. Abdominal computed tomography scan (CT) demonstrated an 8.7 cm × 7.1 cm × 9.5 cm tumor behind the left peritoneum. In urine catecholamines analysis, free-norepinephrine (f-NE), free-dopamine (f-DA), free-normetanephrine (f-NMN), free-3-methoxytyramine (f-3MT), vanillylmandelic acid (VMA), and homovanillic acid (HVA) levels were all greater than the normal range for 24 h except free-metanephrine (f-MN) and free-epinephrine (f-E). Based on these findings, we diagnosed her as NB complicated by catecholamine cardiomyopathy manifested by hypertrophic cardiomyopathy (HCM). Oral metoprolol, spironolactone, captopril and amlodipine furosemide, and intravenously injected sodium nitroprusside and phentolamine were employed for treating HT. After the tumor resection, the blood pressure (BP) and urinary catecholamine levels were all restored. After a follow-up of 7 months, echocardiography indicated normalization of ventricular hypertrophy and function.

**Conclusion:**

This is a rare report showing catecholamine cardiomyopathy in NB children. Tumor resection leads to a return to normal of the catecholamine cardiomyopathy manifested as HCM.

## Introduction

Catecholamine-induced cardiomyopathy (CICMP), a rare, devastating, and difficult-to-treat complication of phaeochromocytoma-paraganglioma (PPGL), is common in pheochromocytoma (1), but uncommon in neuroblastoma (NB). PPGL is a catecholamine-producing neuroendocrine tumor arising from extra-adrenal pheochromocytoma in 80%–85% of the adrenal glands or the rest of the autonomic ganglia (2).

NB is one of the most common solid tumors in early childhood accounting for approximately 8% to 10% of all childhood malignancies (3). The prevalence is 11–13 per million children under 15 years of age, 65 per million children under 1 year of age, and 1 per million children between 10 and 14 years of age (4–6). The median age for diagnosis is 18 months, and 90% of which occurs in children under the age of 10 years (7). The location, size, invasion of the tumor, impact of catecholamine release, and symptoms brought on by the paraneoplastic syndrome are the primary determinants of clinical presentations (8). HT occurs in approximately 10% of patients with NB due to extension of the renal pedicle, compression of the renal parenchyma, catecholamine secretion, or activation of the renin-angiotensin system (9). Elevated levels of catecholamine metabolites can be found in 95% of NB patients, and used for the diagnosis, including catecholamines (dopamine [DA], epinephrine [E], norepinephrine [NE]), metanephrines (3-methoxytyramine [3-MT], metanephrine [MN], normetanephrine [NME]) and phenolic acids (vanillylmandelic acid [VMA], homo-vanillic acid [HVA]) (10–14). VMA and HVA are most commonly analyzed in the urine of patients with NB (15, 16).

Here, we present a case of catecholamine cardiomyopathy manifested with HCM in NB, explain the differential diagnosis, and clinical outcome.

## Case description

A girl aged 5 years and 8 months was admitted to our hospital with ventricular hypertrophy. The child gained and lost weight gradually for more than a year. Five months ago, a heart murmur was discovered during a health check, and her transthoracic echocardiography revealed left ventricular hypertrophy without affecting the left heart’s systolic function. Thus, she was diagnosed with nonobstructive hypertrophic cardiomyopathy (HCM), and began treatment with nutritional therapy. About 3 days before admission, she developed shortness of breath, decreased activity tolerance, and an increased heart rate, with no symptoms of cyanosis, dizziness, headache, nausea, or vomiting. She also had no family history of tumor, diabetes, HT, dyslipidemia, or endocrine disorders. The child gained and lost weight gradually for more than a year.

Her height, weight, and body mass index were 98 cm, 14 kg and 14.6 kg/m^2^, respectively. Blood pressure (BP), pulse rate, respiratory frequency, and temperature were 170/135 mmHg, 130 beats/min, 47 beats/min, and 36.1°C. She had tachypnea and a dilated suprasternal fossa. Auscultation reveals attenuated bronchovesicular lung sounds in the right lung’s basal region. A Class III/6 systolic murmur was heard in the 2–4 intercostal space at the left sternal margin. The abdomen was slightly distended, and a mass was felt in the upper abdomen’s middle and left side, 10 cm below the xiphoid process and 2 cm below the left rib.

**Figure 1:**
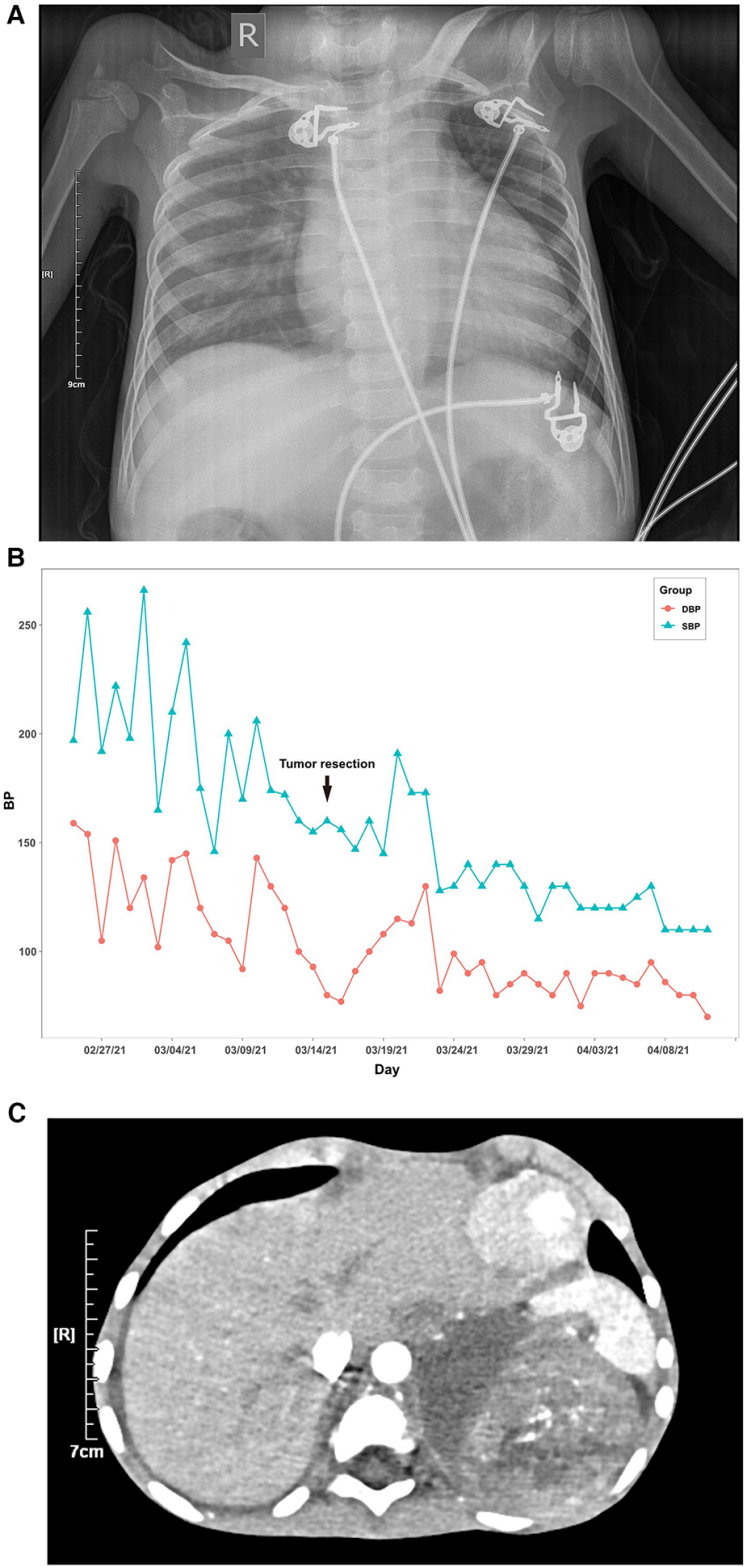
Blood pressure value, chest x-ray and abdominal computed tomography (CT) scan of the patient. (**A**) Line chart of the patient's daily maximum systolic and diastolic blood pressure. (**B**) In chest x-ray, the scattered patches revealed increased density and right pleural effusion, and the cardiac shadow was enlarged. (**C**) In the abdominal computed tomography (CT) scan, there was a right pleural effusion and a soft tissue density mass shadow behind the left peritoneum measuring 8.7 cm × 7.1 cm × 9.5 cm, with a CT value of approximately 28–46 HU. Internal density was varied, with calcified density shadow in certain areas. One possibility is neuroblastoma. The pressure is on the left kidney and spleen.

**Figure 2:**
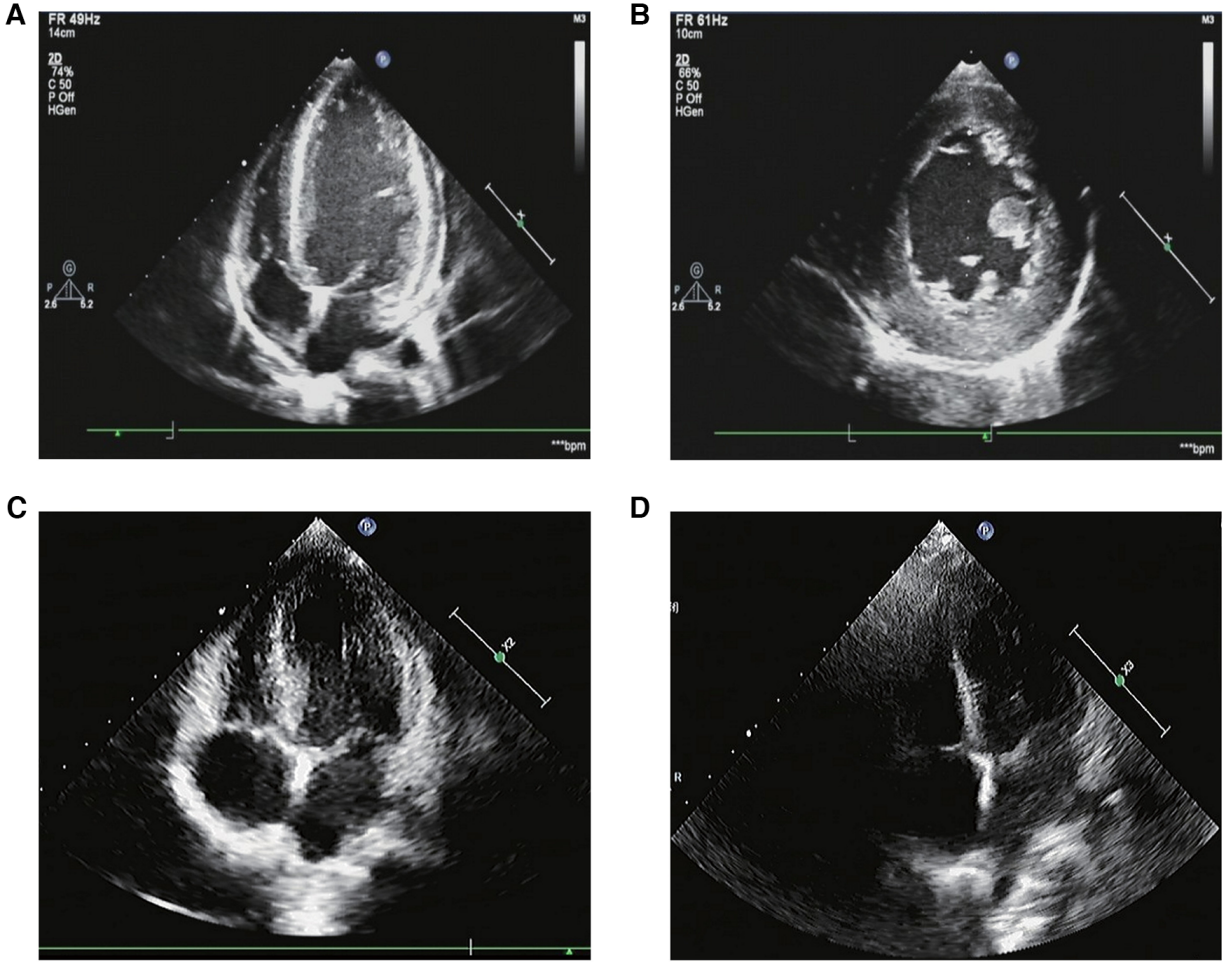
The transthoracic echocardiography. (**A,B**) The transthoracic echocardiography before the tumor resection. (1) The left atrium and ventricle were both enlarged. The ventricular septum and left ventricular free wall were generally thickened. The thicker left ventricular free wall was situated in the middle of the lateral wall, measuring about 14 mm in thickness, while the thicker ventricular septum was situated in the basal segment of the anterior septum, measuring about 12 mm in thickness. The estimated ejection fraction (EF) was about 40%. (2) Both of the bilateral coronary arteries’ internal diameters were increased, with the left main coronary artery’s diameter measuring 4.1 mm and the right main coronary artery’s diameter measuring 3.0 mm. (3) A fluid dark area was observed in the pericardial cavity, 8 mm in the right ventricular sidewall and 4 mm in the apex. (**C**) The transthoracic echocardiography 3 months after the tumor resection. The left ventricle was mildly enlarged. The left ventricular wall was slightly thickened. The thicker left ventricular free wall was situated in the middle of the lateral wall, measuring about 9.5 mm in thickness, while the thicker ventricular septum was situated in the basal segment of the anterior septum, measuring about 10.6 mm in thickness. The systolic function of the left ventricle was normal. (**D**) The transthoracic echocardiography 7 months after the tumor resection. The measured values of the internal diameters of each atrioventricular cavity were normal. Each chamber wall’s thickness and movement were both typical. The systolic function of the left ventricle was normal.

In myocardial markers, creatine kinase isoenzymes (CK-MB), high-sensitive cardiac troponin T (hs-cTnT), and brain natriuretic peptide (BNP) levels were elevated (Table 1). The ECG revealed a rapid atrial autonomic rhythm, biventricular hypertrophy, ST-T changes, and a prolonged QT interval (Supplementary Figure S1). In chest x-rays and computed tomography (CT) scans revealed an enlarged heart shadow, and the right pleural effusion [Figure 1A, Supplementary Figure S2]. Transthoracic echocardiography revealed that the left atrium and ventricle were both enlarged. Left ventricular ejection fraction (EF) was about 40%, and the ventricular septum and free wall of the left ventricle were thickened in general. The internal diameters of the bilateral coronary arteries were widened, with the left main coronary artery diameter of 4.1 mm and the right main coronary artery diameter of 3.0 mm (Figure 2A,B).

**Table 1:** Laboratory data on admission in this subject.

Myocardial damage markers			Urine catecholamines		
CK (U/L)	39	24–229	f-E (ug/L)	7.67	—
CK-MB (U/L)	39↑	0–25	f-NE (ug/L)	1,361.6	—
hs-cTnT (pg/ml)	22.86↑	0–14	f-DA (ug/L)	3,366.89	—
BNP (pg/ml)	20932↑	<300	f-MN (ug/L)	7.25	—
Endocrinology markers			f-NMN (ug/L)	365.95	—
TSH (uIU/ml)	1.37	0.34–6	f-3MT (ug/L)	462.95	—
TT3 (ng/ml)	1.03↓	1.13–1.89	VMA ((ug/L)	112.19	—
TT4 (ug/dl)	9.18	4.02–13.3	HVA (ug/L)	160.39	—
FT3 (pg/ml)	3.34	1.71–4.87	24hUV (ml)	873	—
FT4 (ng/dl)	0.99	0.7–1.48	24h-f-E (ug/24 h)	6.7	0–20
Cortisol (ug/dl)	16.9	5.00–25.00	24h-f-NE (ug/24 h)	1,188.68↑	0–90
ACTH (pg/ml)	18.5	10.00–185.00	24h-f-DA	2,939.29↑	0–600
LH (mIU/ml)	0.02↓	0.07–2.77	(ug/24 h)		
FSH (mIU/ml)	0.07↓	0.14–5.55	24h-f-MN	6.33	0.0–42.5
Prolactin (ng/ml)	1.21↓	3.1–11.2	(ug/24 h)		
Estradiol (pg/ml)	<10↓	10–49	24h-f-NMN	319.47↑	0.0–57.1
Progesterone (ng/ml)	1.2↑	0–0.99	(ug/24 h)		
Testosterone	<0.45	0.03–0.69	24h-f-3MT	404.16↑	0.0–63.8
(nmol/L)			(ug/24 h)		
HCG-*β* (mIU/ml)	<1.2	0–3	24h-VMA	97.94↑	0.0–10.0
Aldosterone (pg/ml)	515.6↑	98–275	(ug/24 h)		
ET (pg/ml)	48.91	43.22–58.38	24h-HVA (ug/24 h)	140.02↑	0.0-7.5
ANP (pg/ml)	311.11↑	50–150	Cancer markers		
Ang-II (pg/ml)	172.96↑	10–30	AFP (ng/ml)	2.64	0.89–8.78
			CEA (ng/ml)	16.32↑	0-6.2

BNP, brain natriuretic peptide; hs-cTnT, high-sensitive cardiac troponin T; CK, creatine kinase; CK-MB, creatine kinase isoenzymes; TSH stands for thyroid-stimulating hormone; TT3 stands for total triiodothyronine; TT4 stands for total thyroxine; FT3 stands for free triiodothyronine; LH stands for luteinizing hormone; FSH for follicle-stimulating hormone; HCG for human chorionic gonadotropin; ET for endothelin; and ACTH for adrenocorticotropic hormone; LH, luteinizing hormone; FSH, follicle-stimulating hormone; HCG-β, human chorionic gonadotropin-β; ET, endothelin; ANP, atrialnatriureticpeptide; Ang-II, angiotensin-II; f-E, free-epinephrine; f-NE, free-norepinephrine; f-DA, free-dopamine; f-MN, free-metanephrine; f-NMN, free-normetanephrine; f-3MT, free-3-methoxytyramine; VMA, vanillylmandelic acid; HVA, homovanillic acid; 24 h UV, 24 h urine volume; AFP, alpha-fetoprotein; CEA, carcinoembryonic antigen.

Her maximum systolic blood pressure (SBP) surpassed 160 mmHg and her maximum diastolic blood pressure (DBP) surpassed 100mmHg for up to 10 days after admission (Figure 1B). In relevant laboratory tests, her thyroid function, cortisol, and adrenocorticotrophic hormone (ACTH) revealed no abnormality. Aldosterone, atrial-natriuretic-peptide (ANP), and angiotensin-II (Ang-II) levels all increased. Except for f-MN and f-E, catecholamine concentrations in children's urine increased significantly, including f-NE, f-DA, f-NMN, f-MT, VMA, and HVA. Her symptoms, signs, and laboratory tests were all consistent with catecholamine cardiomyopathy diagnosis criteria (17).

**Figure 3:**
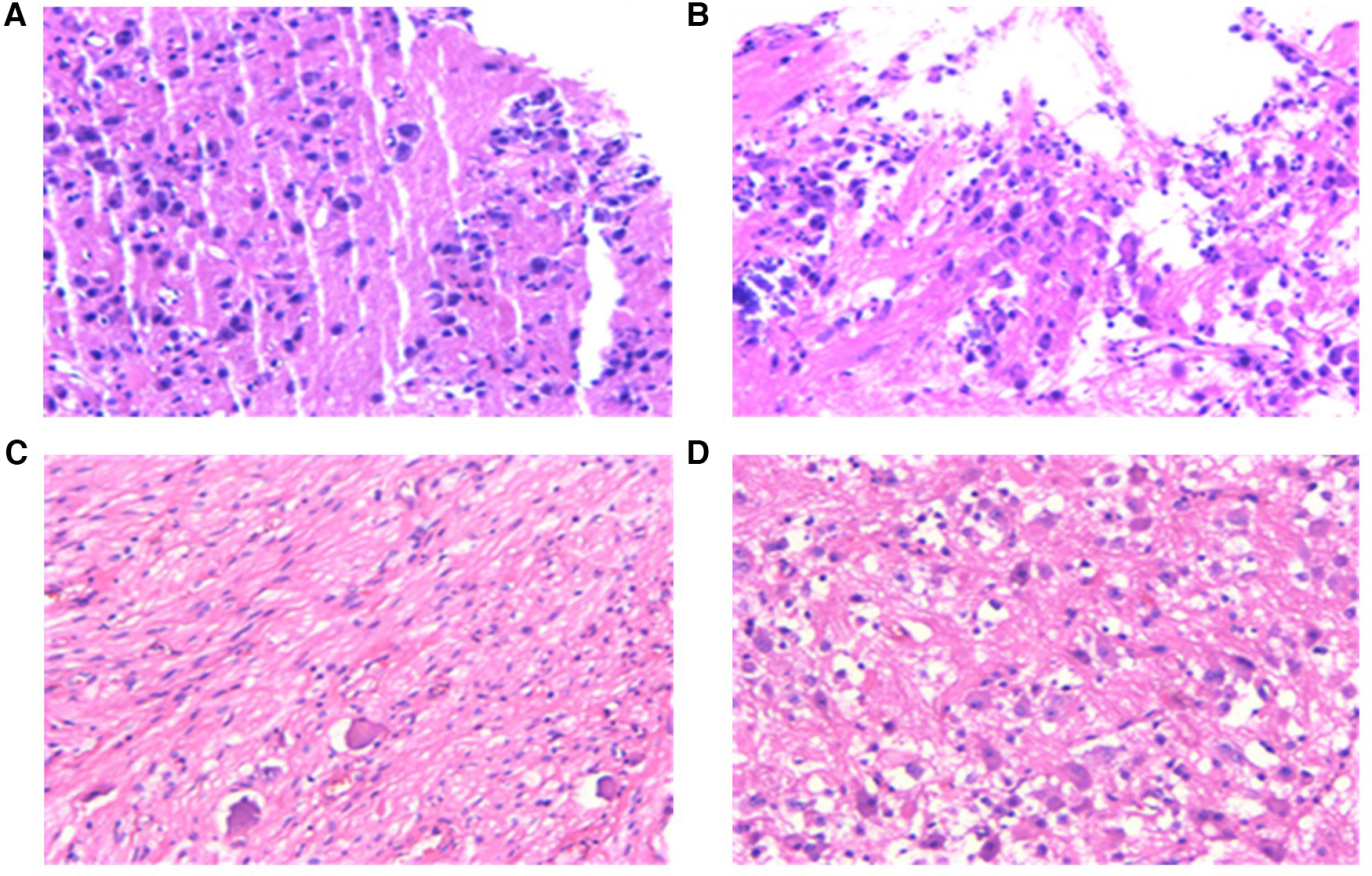
Pathological examination results. (**A,B**), pathology of a puncture biopsy specimen from a retroperitoneal tumor: Neuroblasts distributed in sheets were seen against the background of nerve fibers. The morphology was compatible with developed neuroblastoma with localized calcification, and the pathological diagnosis showed clear extrusion deformation of histiocytes. (**C,D**), pathology of biopsy specimen after surgical resection of tumor: (1) morphology was consistent with neuroblastoma (differentiated) with focal calcification. (2) retroperitoneal adrenal neuroblastoma (differentiated) with focal calcification, MKI < 2%. 3) tumor tissue was found in lymph nodes (aside from renal vein) (6/7). Tumor tissue was found in lymph nodes (aside from the abdominal aorta) (2/2). No tumor cells were discovered in lymph nodes (0/1) and (0/1).

Following the abnormal physical examination of her abdomen, we further performed an abdominal CT examination, which indicated an 8.7 cm × 7.1 cm × 9.5 cm mass shadow with low density behind the left peritoneum (Figure 1C). And her carcinoembryonic antigen (CEA) level was high (Table 1). On the 12th day, the child had a retroperitoneal tumor puncture biopsy. On the 19th day, the left retroperitoneal NB was resected. The detailed pathological findings of biopsy (Figure 3A,B) and resection both supported the diagnosis (Figure 3C,D). As a result, the child was diagnosed with NB complicated by catecholamine cardiomyopathy manifesting as HCM.

The treatment comprised anti-heart failure, anti-hypertension, and surgical therapy. Figure 1A depicts the BP changes during the entire treatment period. Her BP was still considerably raised six hours after admission, and it showed no signs of lowering. Therefore, we stopped the milrinone infusion and oral spironolactone tablets, and kept treating refractory hypertension with the vasodilator sodium nitroprusside and the adrenergic *α* blocker phentolamine for 10 days. During this period, SBP was up to 266 mmHg and DBP was up to 159 mmHg. Amlodipine, in combination with sodium nitroprusside and phentolamine, was used to stabilize BP before tumor resection, followed by captopril and sodium nitroprusside. On the 27th day, we stopped the sodium nitroprusside, and switched to furosemide, spironolactone, metoprolol tartrate, captopril, and amlodipine tablets for 15 days. Then BP was sustained at 110–130/70–100 mmHg, and the values of catecholamines in urine were all returned to normal (Supplementary Table S1). After a subsequent follow-up of 3 months, her transthoracic echocardiography showed that the left ventricular wall became thinner than before, and left ventricular systolic function returned to normal (Figure 2C). After 7 months, her transthoracic echocardiography and hs-cTnT values were restored (Figure 2D). This implied that the catecholamine cardiomyopathy induced severe decline in heart function had been reversed.

## Discussion

This case reported on a child who had catecholamine cardiomyopathy manifested as HCM in the retroperitoneal NB, which was gradually reversible after tumor resection. Most of the reported cases of cardiomyopathy were dilated cardiomyopathy (DCM) with heart failure (18–23), but unusual in NB. HT occurs in about 10% of NB cases (24). Left ventricular hypertrophy is seldom associated with NB (9), and HCM like changes are even rarer, with only 1 reported case in a 4.5-year-old child with sudden death (25). Among the reported DCM cases, urinary catecholamine excretion was elevated in all patients, but HT was present in only a few patients. This proved that myocardial injury and adrenergic receptor downregulation are caused by excessive catecholamine production rather than HT (18–23). This case also demonstrated that, prior to surgery, neither anti-hypertensive nor anti-heart failure treatments were beneficial to the child’s condition. Chronic catecholamine exposure can down-regulate cardiac *β* -adrenergic receptors, resulting in the loss of myofibrils, which results in cardiomyopathy and heart failure (26). Numerous factors contribute to the pathogenesis of cardiomyopathies, such as catecholamine-induced vasoconstriction and coronary vasospasm, chronic tachycardia myopathy brought on by an overactive sympathetic nervous system, adverse adrenergic receptor downregulation, free radical production, and encouragement of calcium influx into the sarcolemma (2, 27).

Catecholamine excess status has been linked to the pathogenesis of multiple cardiomyopathies, including tachycardia-associated cardiomyopathy, HCM, DCM, and Takotsubo cardiomyopathy (TCM) (17,  28–30). The results of chest radiography, electrocardiography, echocardiography, and invasive cardiac studies are crucial in the diagnosis of catecholamine cardiomyopathy (17). In this case, transthoracic echocardiography revealed left atrial and left ventricle enlargement, diffuse thickening of the interventricular septum and left ventricular free wall, and decreased LVEF. The chest radiograph revealed heart enlargement, ECG revealed changes in ST-T and QT interval prolongation, and 24 h urinary catecholamine metabolites were markedly elevated. These all fit the diagnostic criteria for catecholamine cardiomyopathy manifested as HCM. Echocardiography results confirm the distinction between DCM and HCM, with the former exhibiting no significant hypertrophy. Both HCM and DCM can progress to heart failure in the later stages of the disease, which manifests as tachypnea, dyspnea, hyperhidrosis, decreased activity tolerance, pulmonary edema, and reduced EF. As a result, this child began with ventricular hypertrophy and hypertension and progressed to heart failure, which also coincided with the points.

DCM and HCM are treated differently. Angiotensin converting enzyme inhibitors (ACEI), diuretics, digoxin, and nutritional agents have been used in all reported cases of DCM. However, as the loss of left ventricular volume may exacerbate the development of ventricular gradient, diuretics and digoxin should be avoided in HCM. Beta blockers and calcium antagonists, for example, are drugs that promote left ventricular filling. Metoprolol used in this case also controlled catecholamine-induced tachyarrhythmia (31). ACEI, such as captopril, exert the anti-hypertensive effect by directly blocking angiotensin II, and also reduce the severity of intraventricular obstruction and left ventricular hypertrophy (32). Therefore, the level of heart failure, outflow tract obstruction, and renal vascular compression should be taken into account while adjusting anti-hypertensive therapy. However, for catecholamine cardiomyopathy, the most critical treatment remains resection of the catecholamine-secreting NB (33). One of the most important targeted medications used to treat neuroblastoma is anthracyclines, although studies have shown that they have considerable cardiotoxicity (34). It should be initially avoided in this subject. A combination of various chemotherapeutic medications and the proper anti-hypertensive medications could be used to treat the patient’s HT and heart failure in this scenario. Anthracyclines can be considered at a later stage to maximize therapeutic effectiveness.

The child in this case developed coronary dilatation, but certain previous research showed that the coronary usually had no major disease (17, 35, 36). Catecholamine cardiomyopathy and coronary artery dilatation are understudied. Large coronary arteries have a significant proportion of *α* receptors mediating contraction, whereas small coronary arteries are almost entirely equipped with *β* receptors (*β*1 subclass) mediating relaxation (37). Acute excessive adrenergic stimulation causes excessive stimulation of the *β*1 adrenergic receptor, leading to increased cardiac oxygen demand and hypoxia in specific areas, which may contribute to coronary artery vasospasm (38, 39). Thus, we hypothesized that this could be related to coronary artery dilatation.

Catecholamine cardiomyopathy in NB is rare, thus early recognition of the complication is important for diagnosis and treatment. Cardiomyopathy may also occur in the absence of HT due to the direct effect of catecholamine on the myocardium. Therefore, throughout the course of treatment, BP monitoring and cardiovascular assessment should be actively improved, with a focus on cardiac problems connected to NB.

This case remains limited. First, while urine catecholamine metabolites were found, plasma catecholamine metabolites were not. Second, the mechanism of catecholamine cardiomyopathy complicated by coronary artery dilatation requires further investigation. Third, we did not conduct a genetic test on this patient, and it will be refined during a later follow-up to provide more insight into the pathogenesis of catecholamine cardiomyopathy.

## Conclusion

We experienced a case of catecholamine cardiomyopathy in NB manifested with HCM. After tumor resection, catecholamine cardiomyopathy normalized. This case extremely important for clinicians in the early detection and treatment of special cardiomyopathy.

## Data Availability

The raw data supporting the conclusions of this article will be made available by the authors, without undue reservation.
