# Supplementary figures and images for: Case report: Catecholamine cardiomyopathy in children with neuroblastoma
Source: Front Pediatr. 2023 Feb 9;11:1063795. doi: 10.3389/fped.2023.1063795 (PMC9947659; doi:10.3389/fped.2023.1063795)

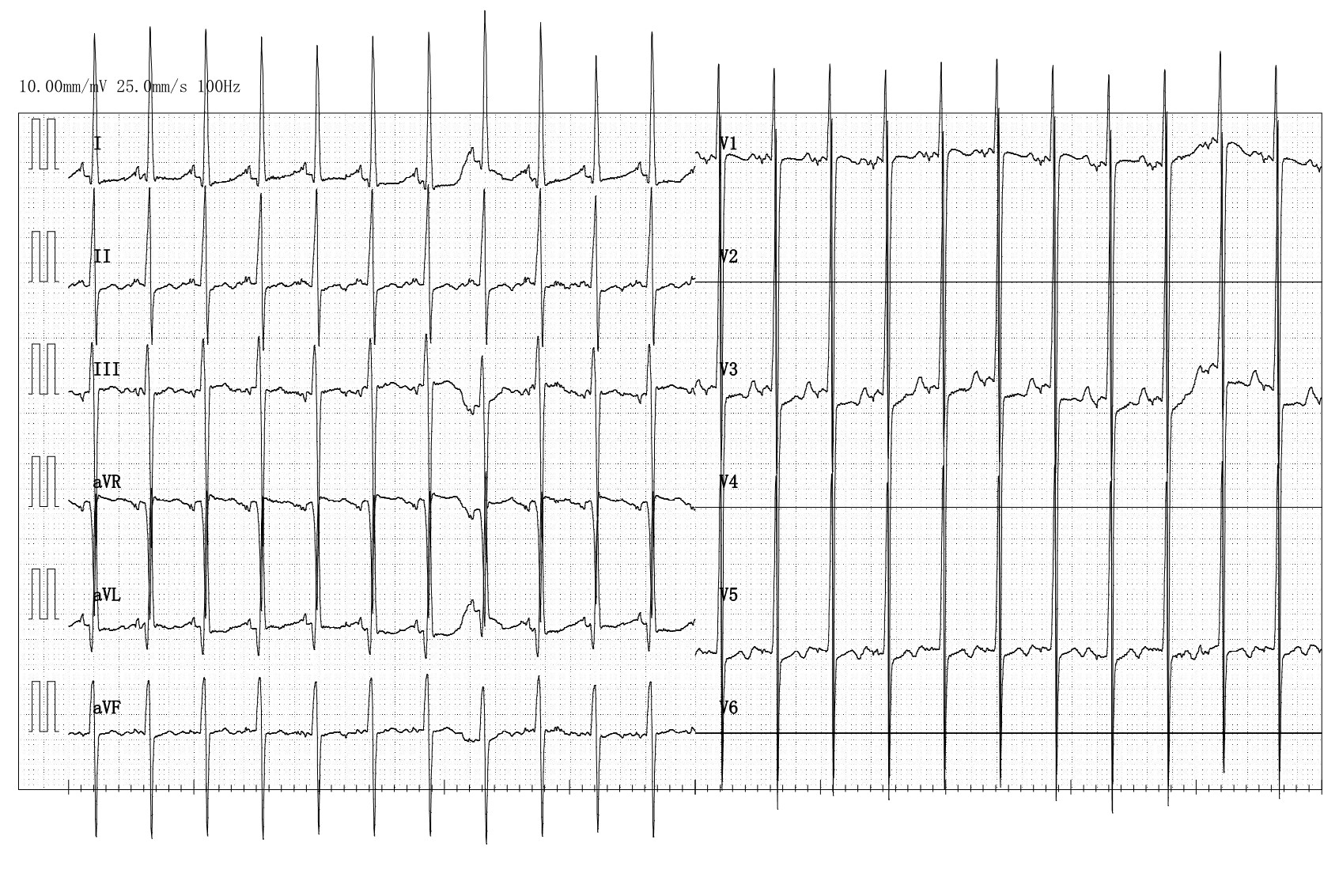

Supplement: Supplementary file 1 [file Image1.jpeg]

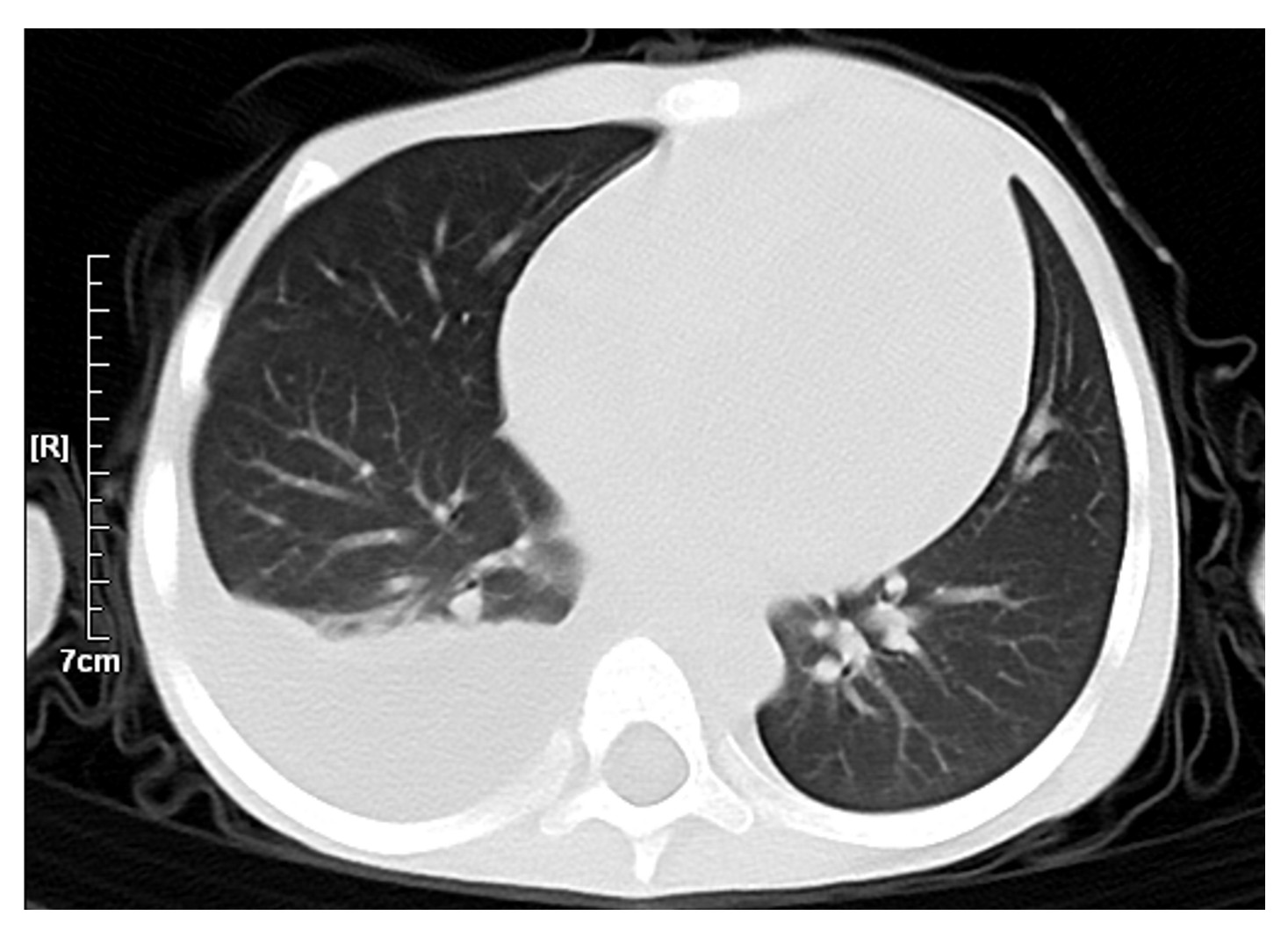

Supplement: Supplementary file 2 [file Image2.jpeg]
