# Supplementary material for: Case report: Catecholamine cardiomyopathy in children with neuroblastoma
Source: Front Pediatr. 2023 Feb 9;11:1063795. doi: 10.3389/fped.2023.1063795 (PMC9947659; doi:10.3389/fped.2023.1063795)
Supplement: Supplementary file 3 [file Table1.docx]

**Table 1**

| **Myocardial damage markers** | | | **Urine catecholamines** | | |
| --- | --- | --- | --- | --- | --- |
| CK (U/L) | 39 | 24-229 | f-E (ug/L) | 7.67 | — |
| CK-MB (U/L) | 39↑ | 0-25 | f-NE (ug/L) | 1361.6 | — |
| hs-cTnT (pg/ml) | 22.86↑ | 0-14 | f-DA (ug/L) | 3366.89 | — |
| BNP (pg/ml) | 20932↑ | <300 | f-MN (ug/L) | 7.25 | — |
| **Endocrinology markers** | | | f-NMN (ug/L) | 365.95 | — |
| TSH (uIU/ml) | 1.37 | 0.34-6 | f-3MT (ug/L) | 462.95 | — |
| TT3 (ng/ml) | 1.03↓ | 1.13-1.89 | VMA ((ug/L) | 112.19 | — |
| TT4 (ug/dl) | 9.18 | 4.02-13.3 | HVA (ug/L) | 160.39 | — |
| FT3 (pg/ml) | 3.34 | 1.71-4.87 | 24hUV (ml) | 873 | — |
| FT4 (ng/dl) | 0.99 | 0.7-1.48 | 24h-f-E (ug/24h) | 6.7 | 0-20 |
| Cortisol (ug/dl) | 16.9 | 5.00-25.00 | 24h-f-NE (ug/24h) | 1188.68↑ | 0-90 |
| ACTH (pg/ml) | 18.5 | 10.00-185.00 | 24h-f-DA | 2939.29↑ | 0-600 |
| LH (mIU/ml) | 0.02↓ | 0.07-2.77 | (ug/24h) |  |  |
| FSH (mIU/ml) | 0.07↓ | 0.14-5.55 | 24h-f-MN | 6.33 | 0.0-42.5 |
| Prolactin (ng/ml) | 1.21↓ | 3.1-11.2 | (ug/24h) |  |  |
| Estradiol (pg/ml) | <10↓ | 10-49 | 24h-f-NMN | 319.47↑ | 0.0-57.1 |
| Progesterone (ng/ml) | 1.2↑ | 0-0.99 | (ug/24h) |  |  |
| Testosterone | <0.45 | 0.03-0.69 | 24h-f-3MT | 404.16↑ | 0.0-63.8 |
| (nmol/L) |  |  | (ug/24h) |  |  |
| HCG-β (mIU/ml) | <1.2 | 0-3 | 24h-VMA | 97.94↑ | 0.0-10.0 |
| Aldosterone (pg/ml) | 515.6↑ | 98-275 | (ug/24h) |  |  |
| ET (pg/ml) | 48.91 | 43.22-58.38 | 24h-HVA (ug/24h) | 140.02↑ | 0.0-7.5 |
| ANP (pg/ml) | 311.11↑ | 50-150 | **Cancer markers** | | |
| Ang-Ⅱ (pg/ml) | 172.96↑ | 10-30 | AFP (ng/ml) | 2.64 | 0.89-8.78 |
|  |  |  | CEA (ng/ml) | 16.32↑ | 0-6.2 |
