# Supplementary material for: Case report: Catecholamine cardiomyopathy in children with neuroblastoma
Source: Front Pediatr. 2023 Feb 9;11:1063795. doi: 10.3389/fped.2023.1063795 (PMC9947659; doi:10.3389/fped.2023.1063795)
Supplement: Supplementary file 4 [file Table2.docx]

**Supplementation legends**

S1. The electrocardiogram of the patient.

1) Accelerated atrial autonomic rhythm. 2) Double chamber hypertrophy. 3) Changes in ST-T. 4) Prolongation of QT interval.

S2. The chest computed tomography (CT) scan.

Right pleural effusion, secondary cardiomegaly, and wall hypertrophy, as well as atelectasis of the right lower lobe.

S-Table 1. Urine catecholamines after tumor resection in this subject.

f-E, free-epinephrine; f-NE, free-norepinephrine; f-DA, free-dopamine; f-MN, free-metanephrine; f-NMN, free-normetanephrine; f-3MT, free-3-methoxytyramine; VMA, vanillylmandelic acid; HVA, homovanillic acid; 24 h UV, 24 h urine volume.
